# Supplementary figures and images for: The Dark Cube: dark character profiles and OCEAN
Source: PeerJ. 2017 Sep 22;5:e3845. doi: 10.7717/peerj.3845 (PMC5611897; doi:10.7717/peerj.3845)

# The Dark Character Cube

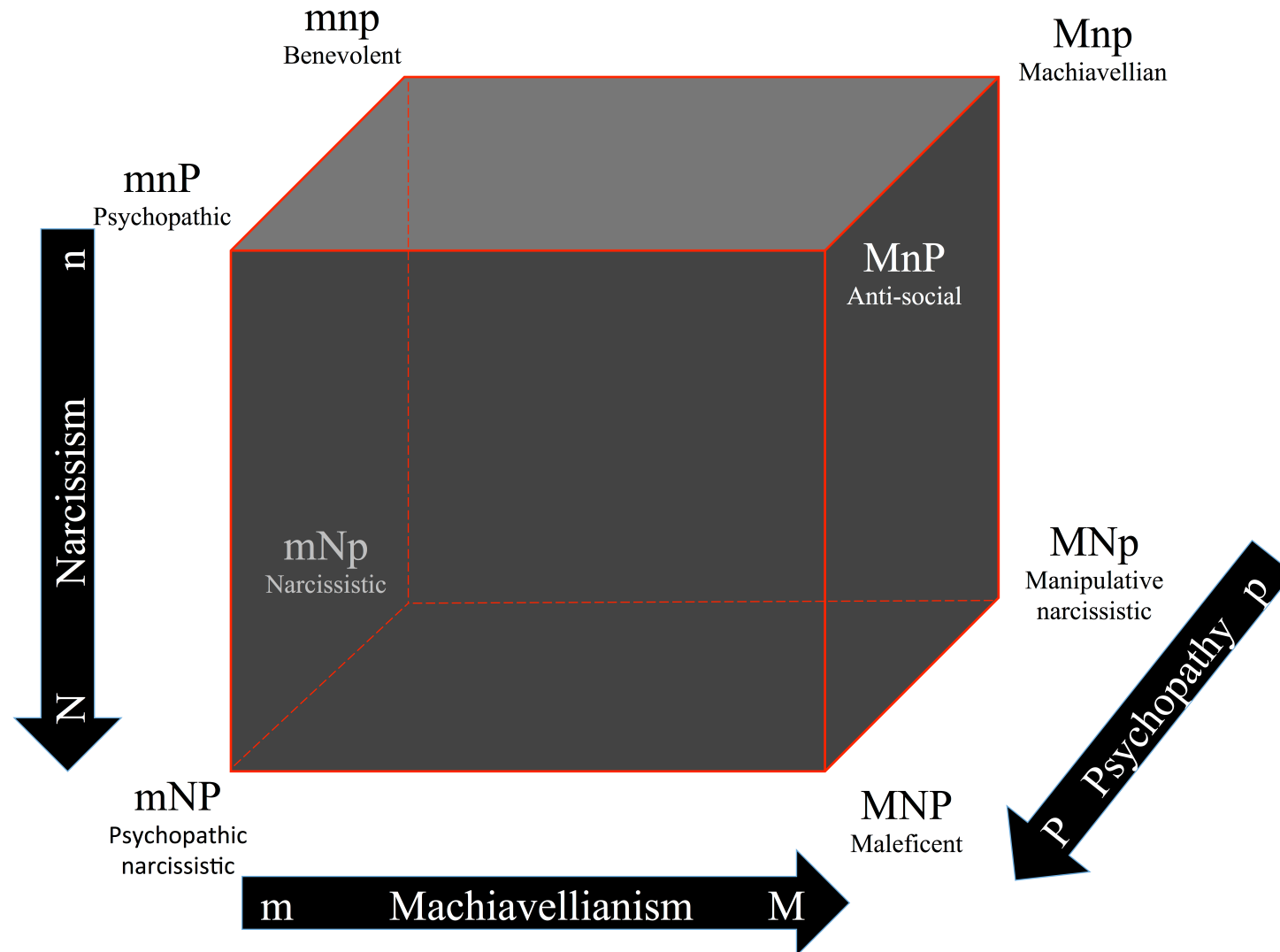

Supplement: Supplemental Information 1 — Note: adapted with permission from CR Cloninger. The directions of the arrows represent higher values. M, high Machiavellianism; m, low Machiavellianism; N, high narcissism; n, low narcissism; P, high psychopathy; p, low psychopathy. Originally published in Garcia D, Rosenberg P (2016). The dark cube: dark and light character profiles. PeerJ 4:e1675. [file peerj-05-3845-s001.pdf]
